# Assessing time-varying causality network of ensemble neural spiking activity

**DOI:** 10.1186/1471-2202-12-S1-P43

**Published:** 2011-07-18

**Authors:** Sanggyun Kim, Marcelo Aguilar, Todd P Coleman

**Affiliations:** 1 Department of Electrical and Computer Engineering, University of Illinois at Urbana-Champaign, Urbana, Illinois 61801, USA; 2 Department of Biological Sciences, Pontifical Catholic University of Chile, Santiago, Chile

## 

Neurons in many brain regions change their spiking responses and interactions among them to relevant stimuli. Tracking the dynamics of neural system is crucial for understanding how neural systems adapt their responses to relevant biological information. Granger causality [1] has been effectively used to assess directional interactions between continuous neural signals, but it cannot be directly applied to neural spike trains viewed as point processes. Recently, methods that extend Granger’s viewpoint to the point process modality have been developed [2], [3] to identify causal interactions between neural spike trains. These methods, however, depend upon stationarity assumptions – which might not be valid when the interactive causal influences themselves are time-varying. We propose a novel probabilistic method for tracking the time-varying causal neural interactions based on sequential prediction of point process models. The time-varying causality from neuron *x* to *y* is assessed by the variability of a windowed version of the point process log-likelihood ratio (LLR), where one model incorporates only the past *y* and the other incorporates the past of both *x* and *y*. The proposed method successfully tracks the time-varying causal network in simulated data, and when applied to real neural data recorded in the rat insular cortex, it identifies the change of causal relationships between neurons to a relevant behavioral stimulus (see Figure 1).

**Figure 1:**
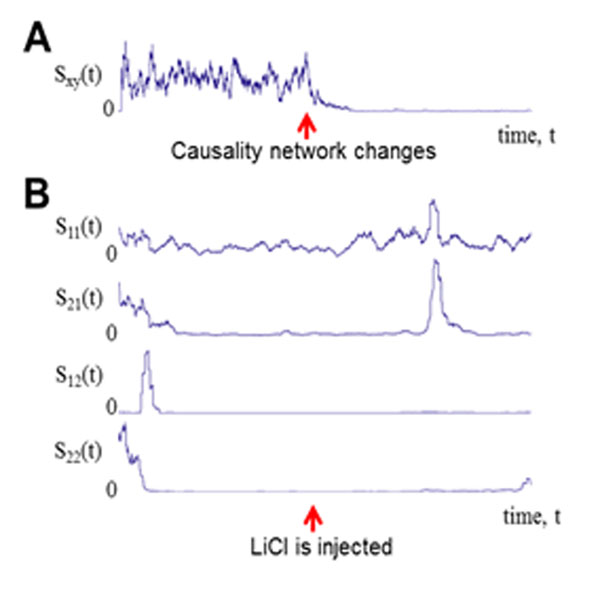
Tracking time-varying causality network. S_ij_(t) represents time-varying causality effect from neuron i to j. **A**. Simulation: Proposed time-varying causality measure had larger values when *x* caused *y* than when *x* did not cause *y*. **B**. Real data analysis: Time-varying causality network was estimated using two neural spike train data recorded in the insular cortex of a rat over before and after LiCl injections.

## Conclusions

The time-varying causal connectivity between neurons was assessed based on the instantaneous point process LLR calculated using the sequential probability assignment method. The proposed framework can be easily extended to different modalities of neural signals using general exponential family of distributions.
